# Comparison of the efficacy between immunochemotherapy and chemotherapy in gastric cancer accompanied with synchronous liver metastases: A real‐world retrospective study

**DOI:** 10.1002/cam4.5917

**Published:** 2023-04-16

**Authors:** Guang‐Tan Lin, Zhi‐Yu Liu, Zhi‐Xin Shang‐Guan, Gui‐Rong Zeng, Jian‐Xian Lin, Ju Wu, Qi‐Yue Chen, Jian‐Wei Xie, Ping Li, Chang‐Ming Huang, Chao‐Hui Zheng

**Affiliations:** ^1^ Department of Gastric Surgery Fujian Medical University Union Hospital Fuzhou China; ^2^ Department of General Surgery Fujian Medical University Union Hospital Fuzhou China; ^3^ Fujian Medical University Diagnostic Pathology Center Fujian Medical University Fuzhou China; ^4^ Key Laboratory of Ministry of Education of Gastrointestinal Cancer Fujian Medical University Fuzhou China; ^5^ Department of General Surgery Affiliated Zhongshan Hospital of Dalian University Dalian China

**Keywords:** gastric cancer, immunotherapy, liver metastases, oncological outcomes, prognosis

## Abstract

**Background:**

Few studies have investigated the efficacy of comprehensive therapies, including immunotherapy, for gastric cancer with synchronous liver metastases (GCLM). We retrospectively compared the effect of immunochemotherapy and chemotherapy alone as conversion therapies on the oncological outcomes of patients with GCLM.

**Methods:**

The clinicopathological data of 100 patients with GCLM from February 2017 to October 2021 at our institution were retrospectively analyzed. Patients were divided into immunochemotherapy (*n* = 33) and chemotherapy‐alone (*n* = 67) groups.

**Results:**

Baseline clinicopathological data did not differ significantly between the two groups. The immunochemotherapy group had a higher overall response rate (59.4% vs. 44.0%, *p* = 0.029) and disease control rate (71.9% vs. 49.2%, *p* = 0.036) than the chemotherapy group. The immunochemotherapy group showed better tumor regression in the gastric mass, metastatic lymph nodes, and liver lesions than the chemotherapy group. Ten (30.3%) patients in the immunochemotherapy group and 13 (19.4%) patients in the chemotherapy group underwent surgery after conversion therapy. However, the difference was not statistically significant. The overall survival (OS) and progression‐free survival (PFS) rates were better in the immunochemotherapy group than in the chemotherapy group. Treatment‐related adverse events occurred in 24 (72.7%) and 47 (70.1%) patients in the immunochemotherapy and chemotherapy groups, respectively.

**Conclusions:**

As a conversion therapy for GCLM, immunotherapy yielded better primary and metastatic tumor regression and survival benefits, with no increase in adverse events compared to chemotherapy.

## Graphical abstract

As a conversion therapy for GCLM, immunotherapy yielded better primary and metastatic tumor regression and survival benefit, with no increase in adverse events than did chemotherapy.

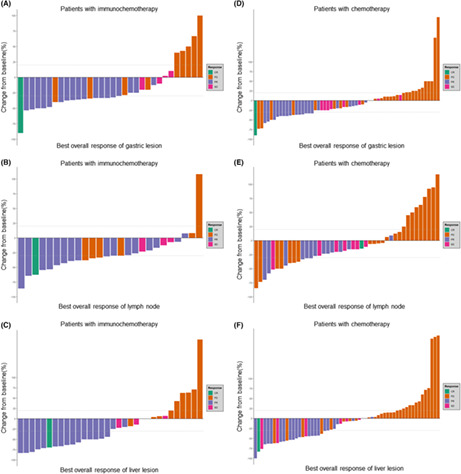

## 1 INTRODUCTION

Gastric cancer (GC) is the fifth most common malignancy and the fourth leading cause of cancer‐related deaths worldwide.^1^ The liver is the most common target organ for hematogenous metastasis of GC, and GC with liver metastasis (GCLM) is one of the main causes of progression and death in advanced GC (AGC).^2^ The incidence rate of GC is approximately 9.9%–18.7%,^3^ of which synchronous GCLM accounts for 73.3%.^4^

The overall prognosis of GCLM is poor, and the current treatment strategies are controversial.^5^, ^6^, ^7^ The surgical resection rate of patients with GCLM is generally low.^8^ In 2016, the REGATTA study confirmed that patients with Stage IV GC with a single non‐curable factor who underwent palliative gastrectomy followed by postoperative chemotherapy did not exhibit significantly better survival than patients who underwent chemotherapy alone.^6^ Notably, most patients with GCLM enrolled in the REGATTA trial had peritoneal metastases. Moreover, no special analysis has exclusively assessed patients with GCLM. Therefore, there are no conclusive guidelines on the surgical indications and choice of resection for GCLM, and disagreements among centers still exist.

It is crucial to develop a conversion therapeutic program with a higher tumor remission rate and lower toxicity to improve the possibility of R0 resection of gastric lesions.^9^, ^10^, ^11^ Wu et al. conducted a Phase II prospective study on induction chemotherapy in patients with GCLM and found that capecitabine plus paclitaxel chemotherapy was effective and safe for improving the overall survival (OS) and resection rates of GCLM.^12^ Li et al. also reported that conversion treatment can improve survival and complete resection rates.^13^ The breakthrough results of immune checkpoint inhibitors also indicate that AGC has entered a new era of immunotherapy, which has led to a paradigm shift in cancer treatment.^14^ The ATTRACTION‐2 trial demonstrated a survival benefit of nivolumab in patients with AGC or gastroesophageal junction cancer.^15^ Moreover, Checkmate‐649 also showed that, compared with chemotherapy alone, nivolumab in combination with chemotherapy showed superior survival benefit and acceptable safety profile in previously untreated patients with AGC. Moreover, in the GCLM subgroup analysis of the study, the median survival time (MST) in patients receiving nivolumab combined with chemotherapy was significantly longer than that in patients receiving chemotherapy alone (nivolumab plus chemotherapy: 12.5 months vs. chemotherapy: 10.6 months).^16^, ^17^

Despite improvements in chemotherapy and molecular biological therapy for AGC,^18^, ^19^ the MST of patients with GCLM receiving chemotherapy is only 10–15 months.^20^, ^21^ Some studies have reported that complete resection of primary GC and GCLM after chemotherapy results in an MST of approximately 26 months and a 5‐year survival rate of 11%–40%.^22^, ^23^, ^24^ Concurrently, with the progress of modern treatment technology and therapeutic concepts, multidisciplinary treatment (MDT) has become the treatment of choice for GCLM. A meta‐analysis found that some highly selected GCLM cases could benefit from conversion therapy and aggressive and timely surgery.^25^

No relevant study has assessed patients with GCLM receiving conversion therapy, including immunotherapy, followed by surgical treatment. Consequently, based on the MDT concept, this study aimed to explore the efficacy, safety, and extent of tumor response to immunotherapy combined with chemotherapy in patients with GCLM and the value of surgical intervention after conversion therapy to provide a high‐level scientific reference for comprehensive treatment of such patients.

## 2 MATERIALS AND METHODS

### 2.1 Study design and cohort

**FIGURE 1:**
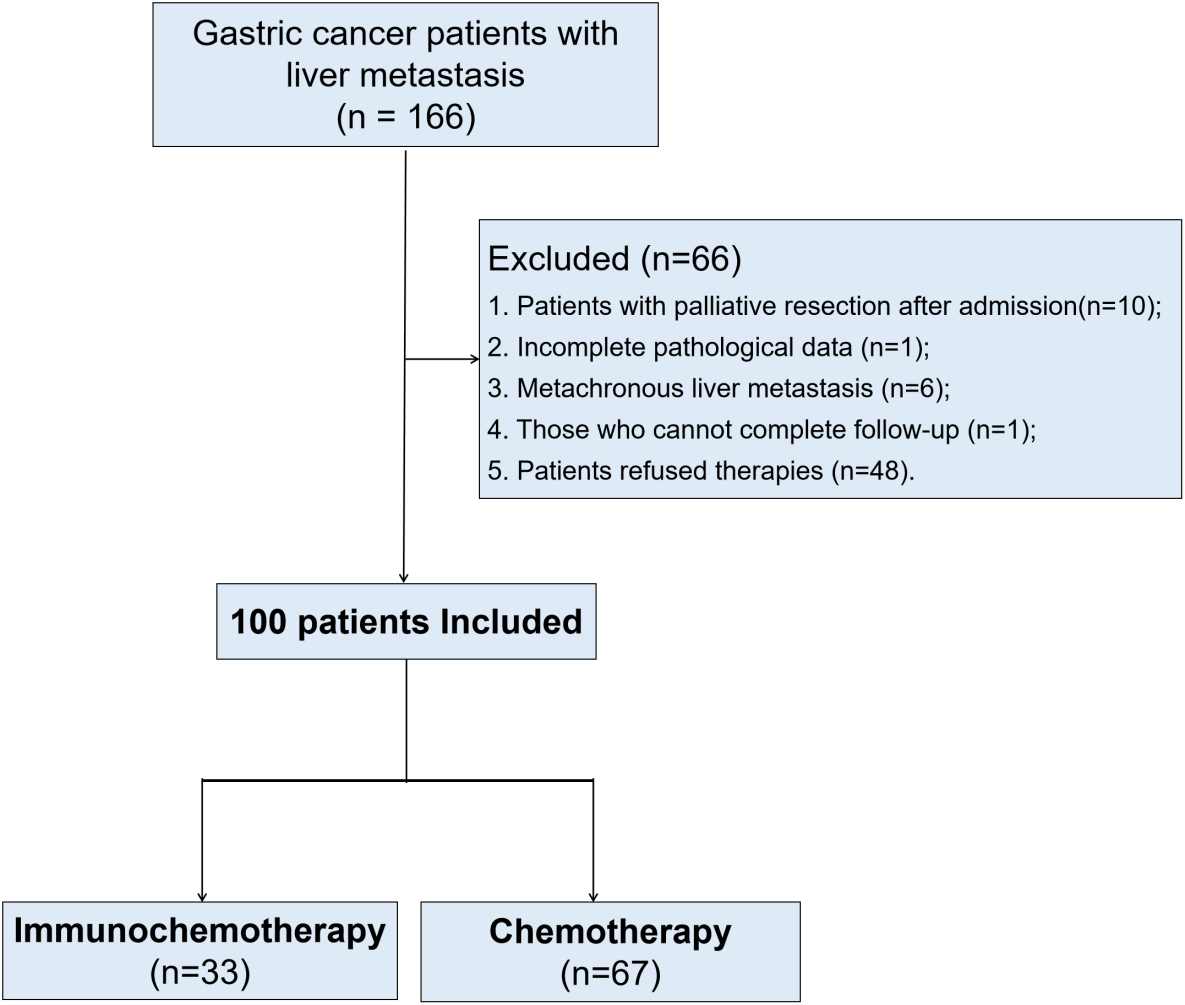
Flow of patient selection.

This retrospective study included 166 patients diagnosed with GCLM between February 2017 and October 2021 at the Department of Gastric Surgery of Fujian Medical University Union Hospital. The inclusion criteria were as follows: pathological diagnosis of GC via surgery/endoscopic biopsy; liver metastasis confirmed on abdominal contrast‐enhanced computed tomography (CT), hepatic magnetic resonance imaging (MRI), positron emission tomography (PET)‐CT, or liver biopsy; and the presence of complete clinical and follow‐up data. The exclusion criteria were as follows: palliative resection after admission, incomplete pathological data, metachronous liver metastasis, missing survival data, and refusal to undergo treatment. Finally, 100 patients with GCLM were included, of whom 33 and 67 were classified into immunochemotherapy and chemotherapy groups, respectively (Figure 1).

### 2.2 Definition and data collection

The extent of lymphadenectomy was determined according to the 2014 Japanese GC treatment guidelines (ver. 4).^26^ GCLM was confirmed in all patients who underwent standard staging procedures for GCLM, the main criteria for which were radiologic findings (CT or MRI of the abdomen) and clinical chemistry profiles. This disease is generally classified into two types: synchronous metastases, defined as metastases occurring before or during surgery or within 6 months after gastrectomy, and metachronous metastases, defined as metastases identified at least 6 months after gastrectomy.^27^ Only patients with synchronous GCLM were included in the study. Cerebral CT or MRI was not part of the routine staging work‐up but was performed in patients in whom the procedure was clinically indicated. Based on these procedures, all tumors were staged according to the 8th American Joint Committee on Cancer staging system. The maximum diameter of the liver metastases and number of liver metastatic lesions were determined using imaging data at the initial diagnosis. In this study, a single metastasis refers only to liver metastasis. Multiple metastases refers to liver metastasis combined with other metastases in other parts of the body, such as the peritoneum, lung, and bone.

### 2.3 Treatment plan

The immunochemotherapy group included patients who received immune checkpoint inhibitors consisting of PD‐1 inhibitors (such as nivolumab, camrelizumab, pembrolizumab, or sintilimab) at least once as conversion therapy. The chemotherapy group included patients who received chemotherapy alone, mainly based on 5‐FU, as a conversion therapy. Patients with HER2 positive receive additional treatment with trastuzumab therapy. Before deciding on the chemotherapy regimen, all patients should be informed of the relevant treatment benefits, risks, and costs, and each patient has the right to choose whether to use PD‐1 inhibitors: immunochemotherapy or chemotherapy alone. Informed consent was obtained from all patients at our center before receiving treatment.

Tumor response was assessed using CT, MRI, or PET‐CT every two to three treatment cycles. The ongoing chemotherapy regimen was continued in patients with stable disease (SD) but was switched to second‐line chemotherapy in patients with progressive disease (PD). Resectability of the gastric primary tumor, tumor activity of the metastatic lymph nodes, and liver metastatic lesions were continuously assessed during conversion therapy. Second‐line chemotherapy regimens were administered to patients with progressive tumors unsuitable for surgical resection.

### 2.4 Evaluation of efficacy outcomes

Tumor response was assessed using Response Evaluation Criteria in Solid Tumors (RECIST), version 1.1^28^ (Tables S1 and S2). The score of tumor response regression score was defined according to the recommendations of the College of American Pathologists as follows: 0 = no viable cancer cells (complete response); 1 = single cells or rare small groups of cancer cells (near complete response); 2 = residual cancer with evident tumor regression, but more than single cells or rare groups of cancer cells (partial response); and 3 = extensive residual cancer with no evident tumor regression (poor or no response). Toxic effects were graded according to the National Cancer Institute Common Terminology Criteria for Adverse Events version 4.0. As this was a retrospective study, the reference for immune‐related adverse events was from previous literature.^29^

The MDT team assessed the tumor resectability based on patient information, including imaging data and individual patient characteristics. The team consisted of two experienced gastrointestinal surgeons: a hepatic surgeon, a gastroenterologist, an interventional physician, an oncologist, a radiotherapy physician, a pathologist, and a radiologist.

### 2.5 Surgery

Currently, there are no guideline recommendations and no multicenter prospective clinical trial data to support the indication of radical surgery for GCLM. According to references and the experiences of our center,^30^ further strategies were determined according to the tumor response to chemotherapy after four to six cycles of conversion chemotherapy. CT was performed to evaluate whether the primary tumor had regressed and was potentially resectable. When liver metastases were deemed stable, shrunken, or even completely disappeared, and the primary gastric tumor could be radically removed, the patient underwent surgery after MDT discussion. The postoperative chemotherapy regimen was determined according to the pathology and gene detection of postoperative specimens and required MDT team discussion; however, it was generally administered no later than 8 weeks after surgery. Morbidity and mortality were assessed within 30 days of surgery. The standard resection for primary gastric lesions is radical gastrectomy + D2 lymph node dissection. Surgical resection criteria for liver metastatic lesions were as follows: 1–3 liver metastases, with the maximum size of the live lesions ≤4 cm (or the lesion is confined to a lobe of the liver), without involving important blood vessels and bile ducts. According to hepatobiliary surgeons, metastatic lesions can achieve R0 resection; radiofrequency ablation can also be used as an auxiliary therapy for surgery or used alone.^31^ Postoperative complications were graded according to the Clavien–Dindo classification. All patients underwent the same perioperative management and follow‐up protocols.

### 2.6 Statistical analysis

According to previous studies, the cutoff values for the maximum diameter of liver metastases and the number of liver metastases were 4 cm and three lesions, respectively.^32^ Using X‐tile software, we found that the optimal cutoff value for the number of conversion therapies to achieve a good OS was three cycles. The OS was calculated from the date of GC diagnosis to death from any cause. Progression‐free survival (PFS) was calculated from the date of GC diagnosis to the first occurrence of disease progression or death from any cause. The follow‐up period was October 2021.

Continuous variables were analyzed using Student's *t*‐test or the Mann–Whitney *U*‐test. We used the chi‐squared test or Fisher's exact test to compare the categorical variables of clinical characteristics. Statistical analyses were performed using SPSS 22.0 (SPSS Inc.) and R software (version 3.5.1). The association between relevant clinicopathological variables and OS was assessed using the Cox proportional hazards model. Stepwise backward variable removal was applied in the multivariate model to identify the most accurate and parsimonious set of predictors. Statistical significance was set at *p* < 0.05.

## 3 RESULTS

### 3.1 General clinicopathological data

Table S3 shows the general clinical data of the immunochemotherapy and chemotherapy‐alone groups. There were no significant differences in age, sex, body mass index, comorbidities, ASA score, performance status, cT stage, cN stage, tumor location and size, surgical resection, maximum size of liver metastatic lesions, number of liver metastatic lesions, Child–Pugh grade, CA724 level, AFP level, CEA level, CA199 level, CA125 level, C‐reactive protein level, albumin level, lymphocyte count level, HER2 status, pathological type, or number of organs with metastasis (all *p* > 0.05). The median follow‐up time was 18 months (range: 1–58).

### 3.2 Tumor response assessment

**FIGURE 2:**
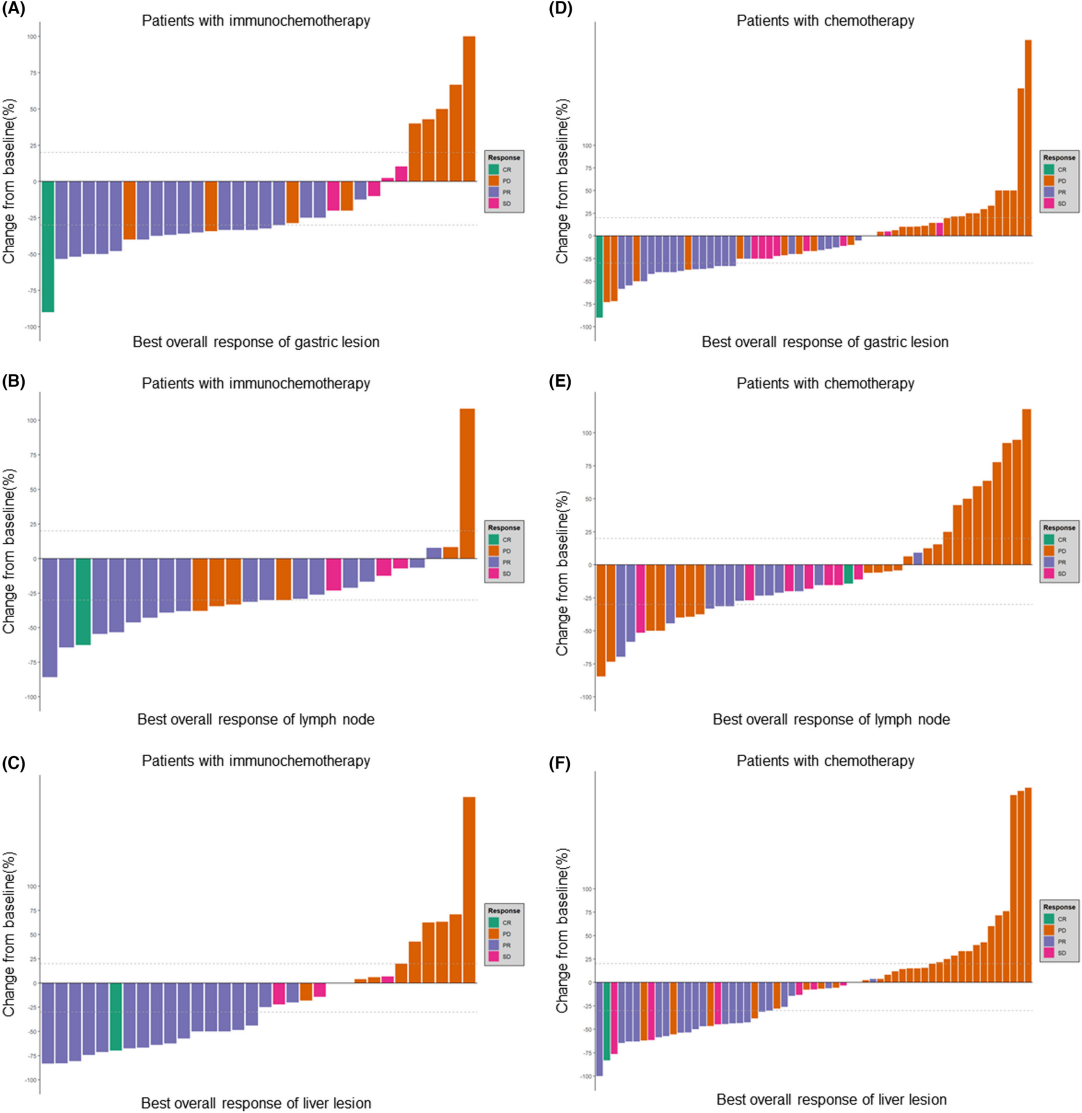
Waterfall plot of the relationship between the overall oncological effect and local lesions. (A) Primary gastric lesion in the immunochemotherapy group; (B) lymph node in the immunochemotherapy group; (C) liver lesion in the immunochemotherapy group; (D) primary gastric lesion in the chemotherapy‐alone group; (E) lymph node in the chemotherapy‐alone group; (F) liver lesion in the chemotherapy‐alone group.

Table 1 shows the overall tumor response of the patients with GCLM receiving treatment; In the whole group, the incidence rates of CR (complete response), PR (partial response), SD, and PD were 2.0% (*n* = 2), 38.0% (*n* = 38), 12.0% (*n* = 12), and 39.0% (*n* = 39), respectively. Nine (9%) patients were not evaluated. The overall response rate (ORR) was 40% (*n* = 40), and the disease control rate (DCR) was 52% (*n* = 52). In the immunochemotherapy group, the CR, PR, SD, and PD incidence rates were 3.0% (*n* = 1), 54.5% (*n* = 18), 12.1% (*n* = 4), and 27.3% (*n* = 9), respectively. One (3%) patient could not be evaluated. In the chemotherapy group, the CR, PR, SD, and PD incidence rates were 1.5% (*n* = 1), 29.9% (*n* = 20), 11.9% (*n* = 8), and 44.8% (*n* = 30), respectively. Moreover, eight (11.9%) patients could not be evaluated. There were significant differences in the ORR (59.4% vs. 44.0%, *p* = 0.029) and DCR (71.9% vs. 49.2%, *p* = 0.036) between the immunochemotherapy and chemotherapy‐alone groups. The immunochemotherapy group showed better overall remission of primary GC, metastatic lymph nodes, and liver metastatic lesions than the chemotherapy‐alone group (Figure 2).

**TABLE 1:** Overall tumor response effects.

Characteristic	Total (*N* = 100)	Immunochemotherapy (*n* = 33)	Chemotherapy (*n* = 67)	*p* Value
CR	2 (2.0)	1 (3.0)	1 (1.5)	**/**
PR	38 (38.0)	18 (54.5)	20 (29.9)	
SD	12 (12.0)	4 (12.1)	8 (11.9)	
PD	39 (39.0)	9 (27.3)	30 (44.8)	
Not evaluable	9 (9.0)	1 (3.0)	8 (11.9)	
Overall response rate**#**	40 (40.0)	19 (59.4)	21 (44.0)	**0.029**
Disease control rate**#**	52 (52.0)	23 (71.9)	29 (49.2)	**0.036**

Data are presented as *N* (%).

Overall response rate = CR + PR; disease control rate = CR + PR + SD.

Nine patients' treatment information was not evaluable.

Abbreviations: CR, complete response; PR, partial response; SD, stable disease. PD, progressive disease.

Bold indicates *p* value < 0.05.

### 3.3 Surgical outcomes

Overall, 23 patients underwent surgical resection, including 10 (30.3%) in the immunochemotherapy group and 13 (19.4%) in the chemotherapy group, with no significant difference (*p* = 0.223). There was no significant difference in the surgical rates between patients with a maximum liver metastasis size of ≤4 and >4 cm (29.2% vs. 17.3%, *p* = 0.159), between patients with less than three metastatic lesions and equal to or more than three liver metastatic lesions (27.0% vs. 11.5%, *p* = 0.106), and between patients with less than two metastatic organs and equal to or more than two metastatic organs (23.1% vs. 22.7%, *p* > 0.999) (Figure S1). All 23 surgical patients received more than three cycles of conversion therapy. There were no significant differences in surgical time, estimated blood loss amount, number of lymph nodes examined, margin, surgical type, liver lesion treatment, ypT, ypN, tumor regression grade, postoperative recovery, complications, and readmission within 30 days between the two groups (all *p* > 0.05) (Table S4).

### 3.4 Survival analysis

**FIGURE 3:**
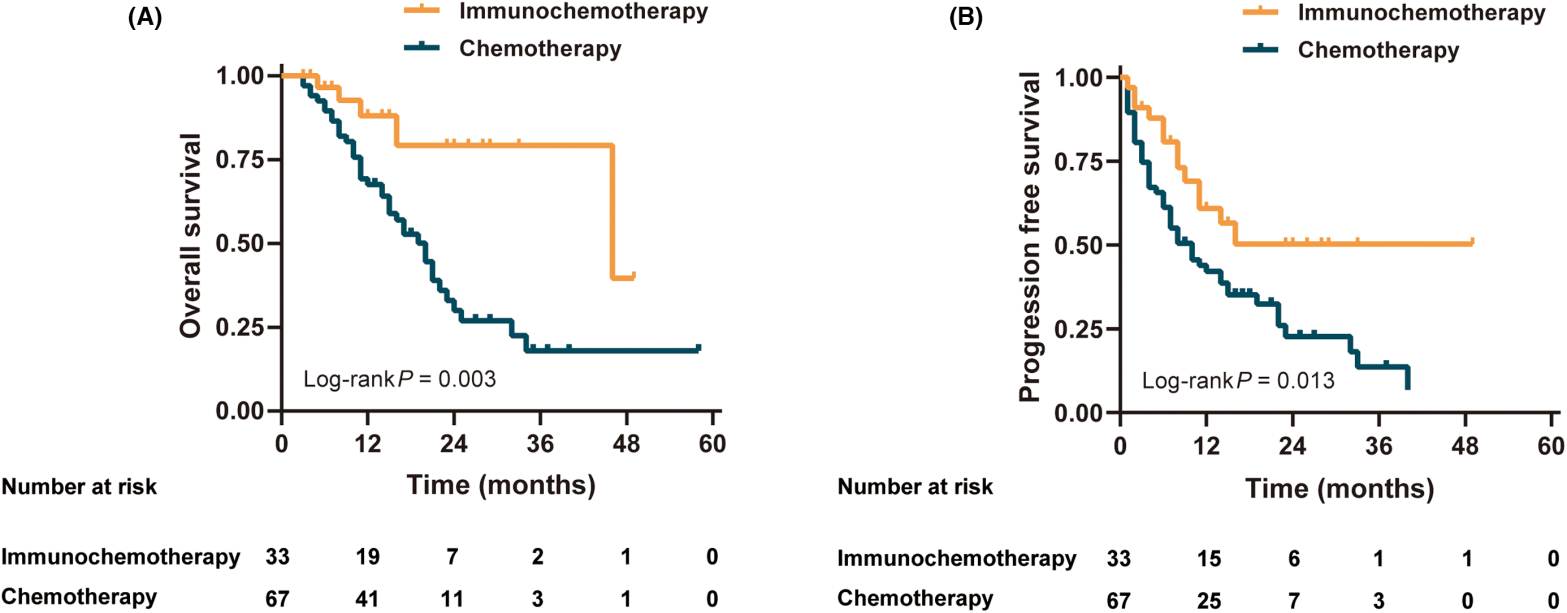
Effects of the different treatments on (A) overall survival and (B) progression‐free survival.

**FIGURE 4:**
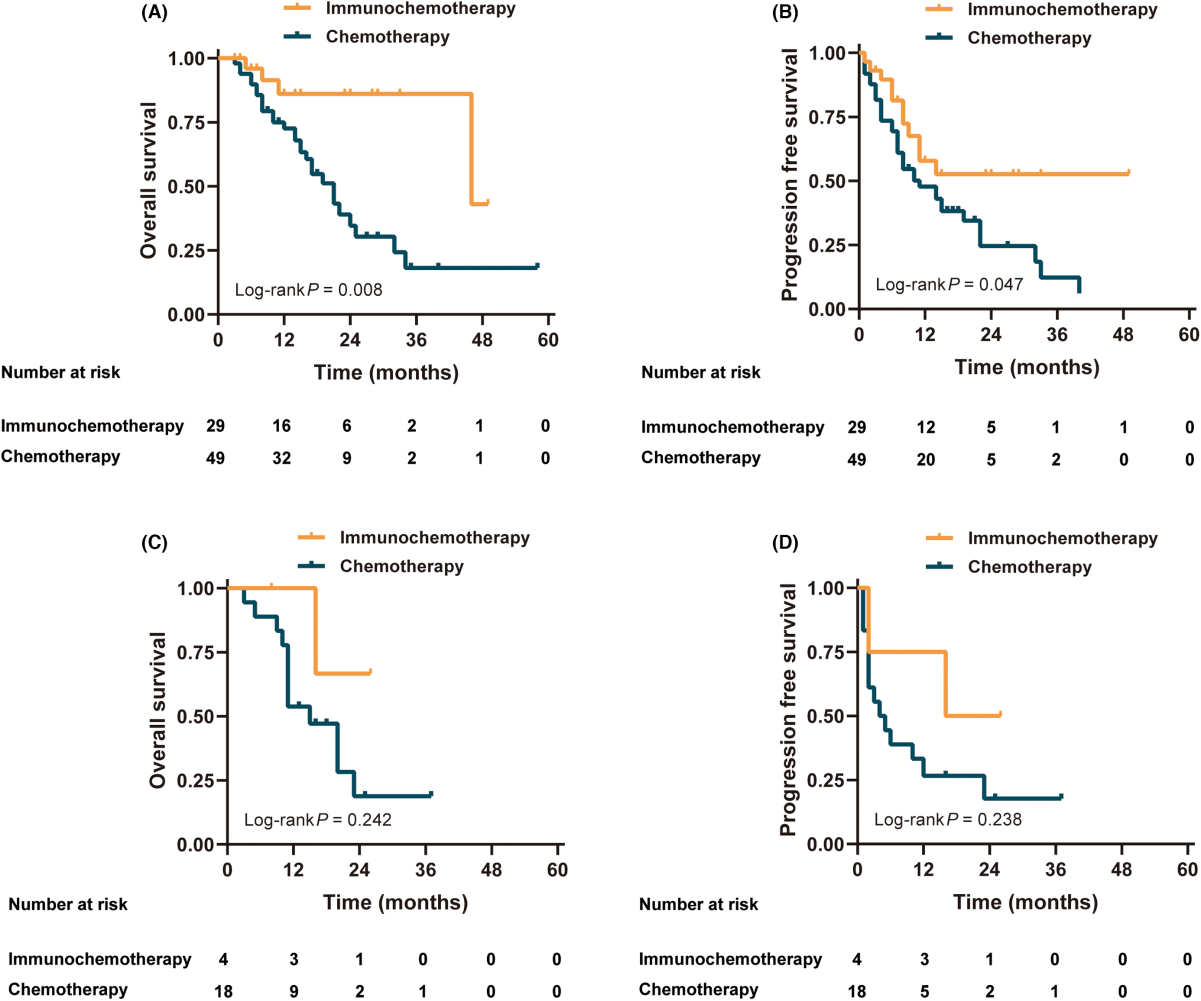
Stratified analysis of the overall survival (OS) and progression‐free survival (PFS) in the overall patients. (A) OS in patients with single metastases. (B) PFS in patients with single metastases. (C) OS in patients with multiple metastases. (D): PFS in patients with multiple metastases.

The survival curve showed that OS and PFS in the immunochemotherapy group were significantly better than those in the chemotherapy group (all *p* < 0.05) (Figure 3). The OS and PFS of patients who underwent surgery were better than those of patients who did not (*p* < 0.05) (Figure S2). Figure 4 shows a stratified analysis of the OS and PFS for liver metastases alone; the immunochemotherapy group had better OS and PFS than the chemotherapy group (*p* = 0.008 and 0.047, respectively). For multiple‐site metastases, the OS and PFS of the immunochemotherapy group were similar to those of the chemotherapy group (*p* = 0.242 and 0.238, respectively). Survival curves (OS and PFS) of patients who receiving ≥3 cycles of conversion therapy were significantly better than those of <3 cycles whether either all patients or subgroups (*p* < 0.05) (Figure S3).

Table S5 shows a comparison of ORR, MST, and median PFS among different studies conducted in patients with AGC receiving immunotherapy. The ORR in the immunochemotherapy group and the Keynote‐062, Checkmate‐649, and Checkmate‐649 subgroups were 59.4%, 48.6%, 58%, and 63%, respectively. The median OS were 12, 12.3, 13.8, and 15.5 months, respectively. The median PFS times were 11, 6.9, 7.7, and 8.5 months, respectively.

### 3.5 Multivariate cox regression analysis

Univariate analysis showed that immunochemotherapy, C‐reactive protein (CRP) level, histological type, surgical resection, maximum size of liver metastatic lesions, and number of conversion therapies were significant factors for OS (all *p* < 0.05). Further multivariate analysis suggested that immunochemotherapy, CRP ≤ 0.5, differentiated histological type, surgical resection, and at least three cycles of conversion therapy were independent protective factors for OS (all *p* < 0.05). Univariate analysis revealed that immunochemotherapy, ASA score, tumor location, surgical resection, and number of conversion therapies were significant factors for PFS (*p* < 0.05). Further multivariate analysis showed that immunochemotherapy and surgical resection were independent protective factors for PFS (all *p* < 0.05) (Table 2).

**TABLE 2:** Cox regression analysis factors associated with the overall survival and progression‐free survival among immunochemotherapy and chemotherapy group.

Clinical parameters	Overall survival								Progression‐free survival							
	Univariable model				Multivariate model				Univariable model				Multivariate model			
	HR	95% CI		*p*	HR	95% CI		*p*	HR	95% CI		*p*	HR	95%CI		*p*
Treatment																
Chemotherapy	Ref				Ref				Ref				Ref			
Immunochemotherapy	0.27	0.11	0.69	**0.006**	0.23	0.09	0.62	**0.003**	0.48	0.26	0.88	**0.017**	0.46	0.25	0.86	**0.015**
Age, years																
<65	Ref								Ref							
≥65	1.44	0.78	2.65	0.239					0.93	0.55	1.58	0.795				
Sex																
Female	Ref								Ref							
Male	0.91	0.43	1.90	0.798					0.86	0.47	1.60	0.640				
BMI, kg/m^2^																
<25	Ref								Ref							
≥25	0.67	0.28	1.61	0.369					0.82	0.43	1.58	0.559				
ASA scores																
1	Ref								Ref							
2	0.93	0.38	2.27	0.874					0.48	0.25	0.90	**0.023**				
3	1.99	0.73	5.40	0.177					1.07	0.50	2.31	0.854				
C‐reactive protein, mg/dL																
>0.5	Ref				Ref				Ref							
≤0.5	0.42	0.22	0.82	**0.011**	0.41	0.20	0.83	**0.013**	1.49	0.87	2.55	0.146				
Immune‐related adverse events																
No	Ref								Ref							
Yes	0.23	0.03	1.68	0.148					0.73	0.29	1.82	0.495				
Albumin, g/dL																
≤3.5	Ref								Ref							
>3.5	0.55	0.27	1.12	0.101					0.93	0.49	1.77	0.827				
Performance status																
0	Ref								Ref							
1	0.95	0.51	1.75	0.859					0.98	0.58	1.66	0.933				
Lymphocyte count, 1000/μL																
≥1000	Ref								Ref							
<1000	1.00	0.42	2.37	0.998					1.13	0.53	2.37	0.756				
Histological type																
Other/Mix	Ref				Ref				Ref							
Differentiated	0.30	0.11	0.86	**0.024**	0.14	0.04	0.45	**0.001**	1.53	0.55	4.25	0.411				
HER2 status																
Positive	Ref								Ref							
Negative/unknown	1.99	0.77	5.15	0.154					1.34	0.64	2.83	0.437				
Tumor location																
Upper	Ref								Ref							
Middle	0.99	0.40	2.45	0.973					0.87	0.43	1.76	0.704				
Lower	1.56	0.73	3.32	0.252					1.12	0.60	2.08	0.733				
≥2 area	2.42	0.96	6.10	0.060					2.51	1.08	5.80	**0.032**				
Tumor size, cm																
≤5	Ref								Ref							
>5	0.67	0.34	1.30	0.233					1.00	0.58	1.73	1.000				
Surgical resection																
No	Ref				Ref				Ref				Ref			
Yes	0.16	0.05	0.52	**0.002**	0.14	0.04	0.50	**0.003**	0.32	0.15	0.67	**0.003**	0.31	0.14	0.66	**0.015**
Maximum size of the liver metastatic lesions, cm																
≤4	Ref								Ref							
>4	2.09	1.14	3.84	**0.017**					1.65	0.99	2.74	0.055				
Number of the liver metastatic lesions																
≤3	Ref								Ref							
>3	1.53	0.82	2.85	0.183					1.45	0.84	2.49	0.179				
Number of conversion therapy																
<3	Ref				Ref				Ref							
≥3	0.26	0.14	0.49	**<0.001**	0.38	0.20	0.72	**0.003**	0.51	0.30	0.87	**0.014**				
Number of organs with metastasis																
<2	Ref								Ref							
≥2	1.51	0.79	2.89	0.214					1.52	0.86	1.00	0.152				

Abbreviation: HER2, human epidermal growth factor 2.

Bold indicates *p* value < 0.05.

### 3.6 Adverse events

Treatment‐related adverse events are shown in Table 3. These adverse events occurred in 24 (72.7%) patients in the immunochemotherapy group and 47 (70.1%) patients in the chemotherapy group, including Grade 3–4 adverse events that occurred in 14 of 33 (42.4%) patients in the immunochemotherapy group and 28 of 67 (41.8%) patients in the chemotherapy‐alone group. Nine patients experienced immune‐related adverse events. No Grade 5 treatment‐related adverse events were observed in either of the groups. The most common treatment‐related adverse events in the immunochemotherapy and chemotherapy‐alone groups were nausea (39.4% vs. 38.8%), neutropenia (30.3% vs. 29.9%), and anemia (24.2% vs. 25.4%). No significant differences were found in fatigue, vomiting, diarrhea, abnormal hepatic function, decreased appetite, peripheral sensory neuropathy, thrombocytopenia, pruritus, stomatitis, or palmar–plantar erythrodysesthesia syndrome.

**TABLE 3:** Adverse events reported.

Event, *N* (%)	All grade	Grades 3–5^a^	All grade	Grades 3–5^a^
	Immunochemotherapy (*n* = 33)		Chemotherapy (*n* = 67)	
Adverse event				
Any adverse event	24 (72.7)	14 (42.4)	47 (70.1)	28 (41.8)
Immune‐related adverse events	9 (27.3)		/	
Any‐grade events of treated patients in either group				
Nausea	13 (39.4)	2 (6.1)	26 (38.8)	2 (3.0)
Neutropenia	10 (30.3)	5 (15.2)	20 (29.9)	11 (16.4)
Anemia	8 (24.2)	2 (6.1)	17 (25.4)	5 (7.5)
Fatigue	7 (21.2)	1 (3.0)	13 (19.4)	2 (3.0)
Vomiting	7 (21.2)	1 (3.0)	15 (22.4)	2 (3.0)
Diarrhea	7 (21.2)	1 (3.0)	15 (22.4)	2 (3.0)
Abnormal hepatic function	7 (21.2)	1 (3.0)	9 (13.4)	2 (3.0)
Decreased appetite	5 (15.2)	1 (3.0)	9 (13.4)	2 (3.0)
Peripheral sensory neuropathy	4 (12.1)	1 (3.0)	8 (11.9)	2 (3.0)
Thrombocytopenia	3 (9.1)	1 (3.0)	6 (9.0)	2 (3.0)
Pruritus	3 (9.1)	0 (0.0)	2 (3.0)	0 (0.0)
Stomatitis	2 (6.1)	1 (3.0)	4 (6.0)	1 (1.5)
PPE syndrome	2 (6.1)	1 (3.0)	4 (6.0)	1 (1.5)

Abbreviation: PPE, palmar‐plantar erythrodysesthesia.

^a^ No Grade 5 adverse events occurred in either group.

## 4 DISCUSSION

The liver is the main target organ for the hematogenous metastasis of GC. Shitara et al. summarized 67 randomized clinical trials, including 12,656 patients with AGC, and found that the incidence rate of liver metastasis was 44%.^33^ Surgical resection is extremely limited in patients with GCLM, and the long‐term prognosis of these patients is poor, with a long‐term survival rate of only approximately 10%.^34^

In this study, we compared the oncological efficacy of immunochemotherapy and chemotherapy in patients with synchronous GCLM. The results confirmed that the overall efficacy against GCLM, ORR, and DCR in patients who received immunotherapy was significantly better than that in patients who received chemotherapy alone. In 2018, Bando et al. conducted a Phase II study on paclitaxel combined with ramucirumab and found that the ORR of patients with advanced unresectable GC who received immunotherapy combined with chemotherapy reached 54.8%.^35^ This finding is similar to that of the present study. Concurrently, in terms of safety, the rate of side effects among the patients receiving immunochemotherapy did not increase significantly compared with that among the patients receiving chemotherapy alone, which is consistent with previous reports that immunochemotherapy does not increase the drug burden on patients and is well tolerated.^15^, ^16^, ^36^ In this study, 54.5% of patients achieved PR after immunochemotherapy, suggesting that this regimen may be effective and can be used for postoperative or palliative care. For patients with potentially resectable GCLM, the recommended treatment is conversion therapy,^37^ which allows patients to undergo resection. After conversion therapy, the size of the primary tumor and metastases can be significantly reduced and the resection rate can be improved. Notably, not all patients who underwent radical resection showed a significant remission response, and some achieved only a mild response (SD). Therefore, it is necessary to investigate the relationship between the tumor response and radical resection to achieve good survival in the future.

In this study, patients who received immunochemotherapy had significantly better OS and PFS rates than those who received chemotherapy alone. Further analysis showed that among patients with liver metastases only, those receiving immunochemotherapy had better OS and PFS than those receiving chemotherapy alone. Simultaneously, immunochemotherapy and surgical treatment were independent protective factors for prognosis, indicating that surgical resection after immunochemotherapy could improve the survival of such patients. The wider the spread, the poorer the prognosis. An appropriate systemic treatment regimen can achieve tumor downstaging and micrometastasis killing, thereby increasing the likelihood of radical resection of the primary tumor. As a novel method, immunotherapy can rapidly attack and effectively shrink target lesions so that additional or better therapeutic options can be explored or implemented. Gastric tumor resection can greatly reduce the macroscopic and potentially immunosuppressive tumor burden, eliminate the source of new metastases, and improve the symptoms caused by gastric injury, thereby further promoting the efficacy of immunotherapy and chemotherapy.^38^ It is noteworthy that low levels of CRP are an independent protective factor for improving OS. Since Virchow first discovered the relationship between inflammation and cancer in 1863,^39^ mounting evidence suggests that tumor progression is not only related to the intrinsic properties of tumor cells but also closely linked to the body's inflammatory immune response.^40^, ^41^, ^42^, ^43^ Our center's previous research has confirmed the value of CRP as a prognostic indicator for GC patients.^44^, ^45^ Additionally, Sato et al. reported an independent correlation between CRP and survival rates of GC patients treated with immune checkpoint inhibitors. The relationship between hematological indicators and the prognosis of GC patients has received widespread attention.^29^ The AIO‐FLOT3 study confirmed that patients who underwent surgical resection (gastrectomy combined with D2 lymph node dissection) after conversion therapy had a better OS than those who received chemotherapy alone (22.9 vs. 10.7 months).^30^ Previous studies also supported our findings.^46^, ^47^, ^48^, ^49^ However, few studies have explored the efficacy of surgical treatment for GCLM after conversion therapy, especially in patients who received PD‐1 inhibitors.^50^, ^51^ Our analysis showed that, although there was no difference in the rate of surgical resection between the two groups, the rate in the immunochemotherapy group was still higher than that in the chemotherapy‐alone group (30.3% vs. 19.4%). This may be because the total number of patients in the immunochemotherapy group was enrolled later than those in the chemotherapy‐alone group, and some patients had not yet reached the expected time point of surgical resection. Granieri et al. conducted a meta‐analysis of the surgical outcomes of GCLM and recommended MDT team discussions for the selection of patients with GCLM to undergo radical resection, which can benefit patient survival.^25^ Moreover, there were no significant differences in the surgical and postoperative short‐term outcomes between the two groups.

This study has several limitations. First, this was a single‐center retrospective study, which may have inherent selection bias, including some patients with distant metastasis other than liver metastasis, response to chemotherapy, and performance status. Such bias might have affected the results. Second, many studies have confirmed that perioperative chemotherapy can improve the surgical resection and survival rates. However, there is no standard chemotherapy strategy for patients with GCLM, which leads to inconsistent chemotherapy regimens. Patients in the immunochemotherapy group did not receive PD‐1 inhibitors in any cycle. Finally, in this study, HER2 data for some patients could not be obtained, and combined positive score measurements were not conducted. However, the ATTRACTION‐2 study showed that the PD‐1 inhibitor group had excellent OS regardless of PD‐L1 expression.^15^ The results of the Phase III clinical study ORIENT‐16 for first‐line treatment of advanced GC showed that among the entire population, patients receiving the combination of XELOX and ramucirumab monotherapy had a prolonged PFS and OS, indicating a benefit. Nevertheless, this is still the first study that systematically reported the long‐term survival outcomes of patients with GCLM who received immunochemotherapy or chemotherapy alone, and found that additional radical gastrectomy after effective conversion therapy can effectively prolong the survival of patients, which can serve as a basis for yielding ideas and provide reference values for further prospective studies in the future.

## 5 CONCLUSION

Immunochemotherapy showed acceptable safety and good oncological efficacy in patients with synchronous GCLM and could significantly improve the long‐term prognosis of such patients. Moreover, immunotherapy combined with chemotherapy can be used to provide an opportunity for surgical resection. In clinical practice, individualized treatment should be administered to patients with synchronous GCLM.

## AUTHOR CONTRIBUTIONS

**Guang‐Tan Lin:** Writing – review and editing (lead). **Zhi‐Yu Liu:** Writing – original draft (lead). **Zhi‐Xin Shang‐Guan:** Software (equal). **Gui‐Rong Zeng:** Visualization (lead). **Jianxian Lin:** Resources (lead). **Ju Wu:** Formal analysis (lead). **Qi‐yue Chen:** Methodology (lead). **Jian‐Wei Xie:** Writing – review and editing (lead). **Ping Li:** Supervision (lead). **Chang‐Ming Huang:** Conceptualization (lead). **Chao‐Hui Zheng:** Project administration (lead).

## FUNDING INFORMATION

This work was supported by the Joint Funds for the Innovation of Science and Technology, Fujian Province (2021Y9042). The support funds for Fujian Province Medical “Creating high‐level hospitals, high‐level medical centers, and key specialty projects (MWYZ [2021] No. 76).

## CONFLICT OF INTEREST STATEMENT

There are no conflicts of interest or financial ties to disclose from any of the authors.

## ETHICS STATEMENT

The study was approved by the Institutional Review Board at the Fujian Medical University Union Hospital. The data obtained after obtaining consent were approved by the scientific research project (ethics approval number 2022KY161).

## CONSENT TO PARTICIPATE

Informed consent was obtained from all the participants included in the study.

## Supporting information


**Supplementary material S1:** **Table S1** Time point response: patients with target and non‐target diseases (Eisenhauer et al.).
**Table S2** Best overall response when confirmation of CR and PR is required (Eisenhauer et al.).
**Table S3** Demographic and baseline characteristics of the two groups.
**Table S4** Surgical and postoperative outcomes.
**Table S5** Comparison among different studies of tumor response and prognosis.
**Figure S1.** Surgical conversion rate for the different groups.
**Figure S2.** Prognosis of different conversion therapies and surgery. (A) Overall survival; (B) progression‐free survival.
**Figure S3.** Survival analysis. Relationship between the number of (A) conversion therapies and OS and (B) conversion therapies and PFS. OS and number of conversion therapies in the (C) immunochemotherapy group and (D) chemotherapy‐alone group. OS, overall survival; PFS, progression‐free survival.

## Data Availability

Data sharing is not applicable to this article as no new data were created or analyzed in this study.
