# Supplementary material for: Comparison of the efficacy between immunochemotherapy and chemotherapy in gastric cancer accompanied with synchronous liver metastases: A real‐world retrospective study
Source: Cancer Med. 2023 Apr 16;12(11):12221–33. doi: 10.1002/cam4.5917 (PMC10278523; doi:10.1002/cam4.5917)
Supplement: Supplementary file 1 — Supplementary material S1: Table S1 Time point response: patients with target and non‐target diseases (Eisenhauer et al.). Table S2 Best overall response when confirmation of CR and PR is required (Eisenhauer et al.). Table S3 Demographic and baseline characteristics of the two groups. Table S4 Surgical and postoperative outcomes. Table S5 Comparison among different studies of tumor response and prognosis. Figure S1. Surgical conversion rate for the different groups. Figure S2. Prognosis of different conversion therapies and surgery. (A) Overall survival; (B) progression‐free survival. Figure S3. Survival analysis. Relationship between the number of (A) conversion therapies and OS and (B) conversion therapies and PFS. OS and number of conversion therapies in the (C) immunochemotherapy group and (D) chemotherapy‐alone group. OS, overall survival; PFS, progression‐free survival. [file CAM4-12-12221-s001.docx]

**Supplementary Online Content**

**eTable 1** Time point response: patients with target and non-target diseases. (Eisenhauer et al.)

**eTable 2** Best overall response when confirmation of CR and PR is required (Eisenhauer et al.)

**eTable 3** Demographic and baseline characteristics of the two groups

**eTable 4** Surgical and postoperative outcomes

**eTable 5** Comparison among different studies of tumor response and prognosis

**eFigure 1.** Surgical conversion rate for the different groups

**eFigure 2.** Prognosis of different conversion therapies and surgery. A: overall survival; B: progress free survival.

**eFigure 3.** Survival analysis. A: Relationship between the number of conversion therapies and OS; B: Relationship between the number of conversion therapies and PFS. C: OS and number of conversion therapies in the immunochemotherapy group. D: OS and number of conversion therapies in the chemotherapy alone group (OS: overall survival, PFS: Progress free survival)

**eTable 1**. Time point response: patients with target and non-target disease. (Eisenhauer *et al.*)

| Target lesions a | Non-target lesions b | New lesions c | Overall  response |
| --- | --- | --- | --- |
| CR | CR | No | CR |
| CR | Non-CR/non-PD | No | PR |
| CR | Not evaluated | No | PR |
| PR | Non-PD or not all evaluated | No | PR |
| SD | Non-PD or not all evaluated | No | SD |
| Not all evaluated | Non-PD | No | NE |
| PD | Any | Yes or No | PD |
| Any | PD | Yes or No | PD |
| Any | Any | Yes | PD |

**a: Complete Response (CR)**: Disappearance of all target lesions. Any pathological lymph nodes (whether target or non-target) must have reduction in short axis to <10 mm. **Partial Response (PR):** At least a 30% decrease in the sum of diameters of target lesions, taking as reference the baseline sum diameters. **Stable Disease (SD):** Neither sufficient shrinkage to qualify for PR nor sufficient increase to qualify for PD, taking as reference the smallest sum diameters while on study. **Progressive Disease (PD):** At least a 20% increase in the sum of diameters of target lesions, taking as reference the smallest sum on study (this includes the baseline sum if that is the smallest on study). In addition to the relative increase of 20%, the sum must also demonstrate an absolute increase of at least 5 mm. (Note: the appearance of one or more new lesions is also considered progression). **Inevaluable (NE)**.

**b: CR**: Disappearance of all non-target lesions and normalisation of tumour marker level. All lymph nodes must be non-pathological in size (<10 mm short axis). **Non-CR/Non-PD**: Persistence of one or more non-target lesion(s) and/or maintenance of tumour marker level above the normal limits. **PD:** Unequivocal progression (see comments below) of existing non-target lesions. (Note:the appearance of one or more new lesions is also considered progression).

**c:** A lesion identified on a follow-up study in an anatomical location that was not scanned at baseline is considered a new lesion and will indicate disease progression.

**eTable 2**. Best overall response when confirmation of CR and PR required. (Eisenhauer *et al.*)

| Overall response | Overall response | BEST overall response |
| --- | --- | --- |
| First time point | Subsequent time point |  |
| CR | CR | CR |
| CR | PR | SD,PD or PR^a^ |
| CR | SD | SD provided minimum criteria for SD duration met, otherwise, PD |
| CR | PD | SD provided minimum criteria for SD duration met, otherwise, PD |
| CR | NE | SD provided minimum criteria for SD duration met, otherwise NE |
| PR | CR | PR |
| PR | PR | PR |
| PR | SD | SD |
| PR | PD | SD provided minimum criteria for SD duration met, otherwise, PD |
| PR | NE | SD provided minimum criteria for SD duration met, otherwise NE |
| NE | NE | NE |

CR = complete response, PR = partial response, SD = stable disease, PD = progressive disease, and NE = inevaluable.

**a** If a CR is truly met at first time point, then any disease seen at a subsequent time point, even disease meeting PR criteria relative to baseline, makes the disease PD at that point (since disease must have reappeared after CR). Best response would depend on whether minimum duration for SD was met. However, sometimes ‘CR’ may be claimed when subsequent scans suggest small lesions were likely still present and in fact the patient had PR, not CR at the first time point. Under these circumstances, the original CR should be changed to PR and the best response is PR.

**eTable 3** Demographic and baseline characteristics of the two groups

| **Characteristic** | **Mean.(SD) / No.(%)** | | P Value |
| --- | --- | --- | --- |
|  | **Immunochemotherapy (n=33)** | **Chemotherapy (n=67)** |  |
| **Age, y** | 60.0 (9.5) | 62.6 (9.3) | 0.198 |
| **Sex** |  |  | 0.704 |
| Female | 8 (24.2) | 14 (20.9) |  |
| Male | 25 (75.8) | 53(79.1) |  |
| **BMI, kg/m^2^** |  |  | 0.202 |
| <25 | 24 (72.7) | 56 (83.6) |  |
| ≥25 | 9 (27.3) | 11 (16.4) |  |
| **Comorbidity** |  |  | 0.280 |
| No | 21 (63.6) | 35 (52.2) |  |
| Yes | 12 (36.4) | 32 (47.8) |  |
| **ASA scores** |  |  | 0.214 |
| 1 | 7 (21.2) | 7 (10.4) |  |
| 2 | 22 (66.7) | 45 (67.2) |  |
| 3 | 4 (12.1) | 15 (22.4) |  |
| **Performance status** |  |  | 0.256 |
| 0 | 24 (72.7) | 41 (61.2) |  |
| 1 | 9 (27.3) | 26 (38.8) |  |
| **cT stage** |  |  | 0.765 |
| T3 | 2(6.1) | 6 (9.0) |  |
| T4a | 24(72.7) | 50 (74.6) |  |
| T4b | 7(21.2) | 11 (16.4) |  |
| **cN stage** |  |  | 0.681 |
| N0 | 3 (9.1) | 4 (6.0) |  |
| N+ | 30 (90.9) | 63 (94.0) |  |
| **Tumor location** |  |  | 0.460 |
| Upper | 11 (33.3) | 20 (29.9) |  |
| Middle | 11 (33.3) | 14 (20.9) |  |
| Lower | 9 (27.3) | 26 (38.8) |  |
| ≥2 area | 2 (6.1) | 7 (10.4) |  |
| **Tumor size, cm** |  |  | 0.125 |
| ≤5 | 27 (81.8) | 45 (67.2) |  |
| >5 | 6 (18.2) | 22 (32.8) |  |
| **Surgical resection** |  |  | 0.223 |
| No | 23 (71.9) | 54 (80.6) |  |
| Yes | 10 (30.3) | 13 (19.4) |  |
| **Maximum size of the liver metastatic lesions, cm** |  |  | 0.179 |
| ≤4 | 19 (57.6) | 29 (43.3) |  |
| >4 | 14 (42.4) | 38 (56.7) |  |
| **Number of the liver metastatic lesions** |  |  | 0.444 |
| ≤3 | 26 (78.8) | 48 (71.6) |  |
| >3 | 7 (21.2) | 19 (28.4) |  |
| **Child-Pugh grade** |  |  | >0.999 |
| **A** | 32 (97.0) | 64 (95.5) |  |
| **B** | 1 (3.0) | 2 (3.0) |  |
| **C** | 0 (0.0) | 1 (1.5) |  |
| **CA72-4,U/ml** | 12.1 (22.8) | 18.2 (38.7) | 0.407 |
| **AFP,ng/ml** | 186.4 (386.9) | 143.8 (329.0) | 0.567 |
| **CEA,ng/ml** | 69.0 (176.3) | 70.1 (208.0) | 0.979 |
| **CA19-9,U/ml** | 123.3 (265.4) | 166.8 (322.0) | 0.503 |
| **CA125,U/ml** | 58.3 (171.2) | 59.9 (133.2) | 0.958 |
| **C-reactive protein, mg/dL** |  |  | 0.958 |
| ≤0.5 | 12 (36.4) | 24 (35.8) |  |
| >0.5 | 21 (63.6) | 43 (64.2) |  |
| **Albumin, g/dL** |  |  | 0.576 |
| ≤3.5 | 25 (75.8) | 54 (80.6) |  |
| >3.5 | 8 (24.2) | 13 (19.4) |  |
| **Lymphocyte count, 1000/μL** |  |  | 0.536 |
| **≥1000** | 30 (90.9) | 57 (85.1) |  |
| **<1000** | 3 (9.1) | 10 (14.9) |  |
| **HER2 status** |  |  | 0.676 |
| Positive | 6 (18.2) | 10 (14.9) |  |
| Negative/unknown | 27 (81.8) | 57 (85.1) |  |
| **Pathological type** |  |  | 0.201 |
| Adenocarcinoma | 30 (90.9) | 64 (95.5) |  |
| Mix | 3 (9.1) | 1 (1.5) |  |
| Other | 0 (0.0) | 2 (3.0) |  |
| **Number of organs with metastasis** |  |  | 0.094 |
| <2 | 29 (87.9) | 49 (73.1) |  |
| ≥2 | 4 (12.1) | 18 (26.9) |  |
| **Follow-up time, months** | 18 (1-58) | |  |
| **Year** |  |  | - |
| 2017 | 2 (6.1) | 7 (10.4) |  |
| 2018 | 0 (0.0) | 22 (32.8) |  |
| 2019 | 7 (21.2) | 15 (22.4) |  |
| 2020 | 13 (39.4) | 17 (25.4) |  |
| 2021 | 11 (33.3) | 6 (9.0) |  |

Abbreviations: BMI, body mass index (calculated as weight in kilograms divided by height in meters squared); Overlap, Overlapping lesion of stomach; ASA, American Society of Anesthesiologists; **HER2, Human epidermal growth factor 2.**

**eTable 4** Surgical and postoperative outcomes

| **Characteristic** | **Mean.(SD) / No.(%)** | | P **Value** |
| --- | --- | --- | --- |
|  | **Immunochemotherapy (n=10)** | **Chemotherapy (n=13)** |  |
| **Surgical time (minutes)** | 179.0 (63.3) | 210.9 (57.9) | 0.222 |
| **Estimated blood loss (mL)** | 49.5 (19.8) | 49.2 (24.7) | 0.978 |
| **Lymph node examined** | 44.5 (20.8) | 35.9 (13.3) | 0.254 |
| **Margin** |  |  | / |
| Negative | 10 (100.0) | 13 (100) |  |
| Positive | 0 (0.0) | 0 (0.0) |  |
| **Surgical type** |  |  | 0.068 |
| Distal | 3 (30.0) | 0 (0.0) |  |
| Total | 7 (77.8) | 13 (100) |  |
| **Liver lesion treatment** |  |  |  |
| Surgical resection | 1 (10.0) | 0 (0.0) | 0.435 |
| Radiofrequency ablation | 1 (10.0) | 0 (0.0) | 0.435 |
| **ypT** |  |  | 0.306 |
| T0 | 1 (10.0) | 1 (7.7) |  |
| T1 | 2 (20.0) | 0 (0.0) |  |
| T2 | 0 (0.0) | 3 (23.1) |  |
| T3 | 5 (50.0) | 8 (61.5) |  |
| T4a | 1 (10.0) | 1 (7.7) |  |
| T4b | 1 (10.0) | 0 (0.0) |  |
| **ypN** |  |  | 0.221 |
| N0 | 5 (50.0) | 3 (23.1) |  |
| N+ | 5 (50.0) | 10 (76.9) |  |
| **TRG** |  |  | 0.741 |
| 0 | 1 (10.0) | 1 (7.7) |  |
| 1 | 4 (40.0) | 4 (30.8) |  |
| 2 | 3 (30.0) | 7 (53.8) |  |
| 3 | 2 (20.0) | 1 (7.7) |  |
| **Postoperative recovery** |  |  |  |
| Time to ﬁrst ﬂatus (days) | 2.1 (0.6) | 2.3 (0.5) | 0.353 |
| Time to ﬁrst liquid intake (days) | 3.1 (0.6) | 3.4 (0.5) | 0.219 |
| Time to ﬁrst semifluid intake (days) | 5.2 (0.6) | 5.5 (0.9) | 0.315 |
| Postoperative hospital stays (days) | 7.1 (0.6) | 7.7 (1.1) | 0.140 |
| **Postoperative complication** |  |  | 0.486 |
| No | 10 (100.0) | 11 (84.6) |  |
| Yes | 0 (0.0) | 2 (15.4) |  |
| **Postoperative readmission within 30 days** |  |  | 0.486 |
| No | 10 (100.0) | 11 (84.6) |  |
| Yes | 0 (0.0) | 2 (15.4) |  |
| **30 day mortality** | 0 (0.0) | 0 (0.0) | / |

Abbreviations: TRG, tumor regression grade

**eTable 5** Comparison among different studies of tumor response and prognosis

| Characteristic | Toal | Immunochemotherapy | Keynote-062 Pembro+Chemo PD-L1+ | Checkmate-649 Nivo+FOLFOX/XELOX | Checkmete-649 Chinese Subgroup Nivo+FOLFOX/XELOX |
| --- | --- | --- | --- | --- | --- |
|  |  |  | (CPS≥1) |  | (CPS≥5) |
|  | (N=100) | (N=33) | (N=257) | (N=789) | (N=75) |
| **ORR(n=100)** | 40.0% | 59.4% | 48.6% | 58% | 63% |
| **Median OS (months)** | 15 | 12 | 12.3 | 13.8 | 15.5 |
| **mPFS** | 8.5 | 11 | 6.9 | 7.7 | 8.5 |

Abbreviations: ORR, Overall response rate


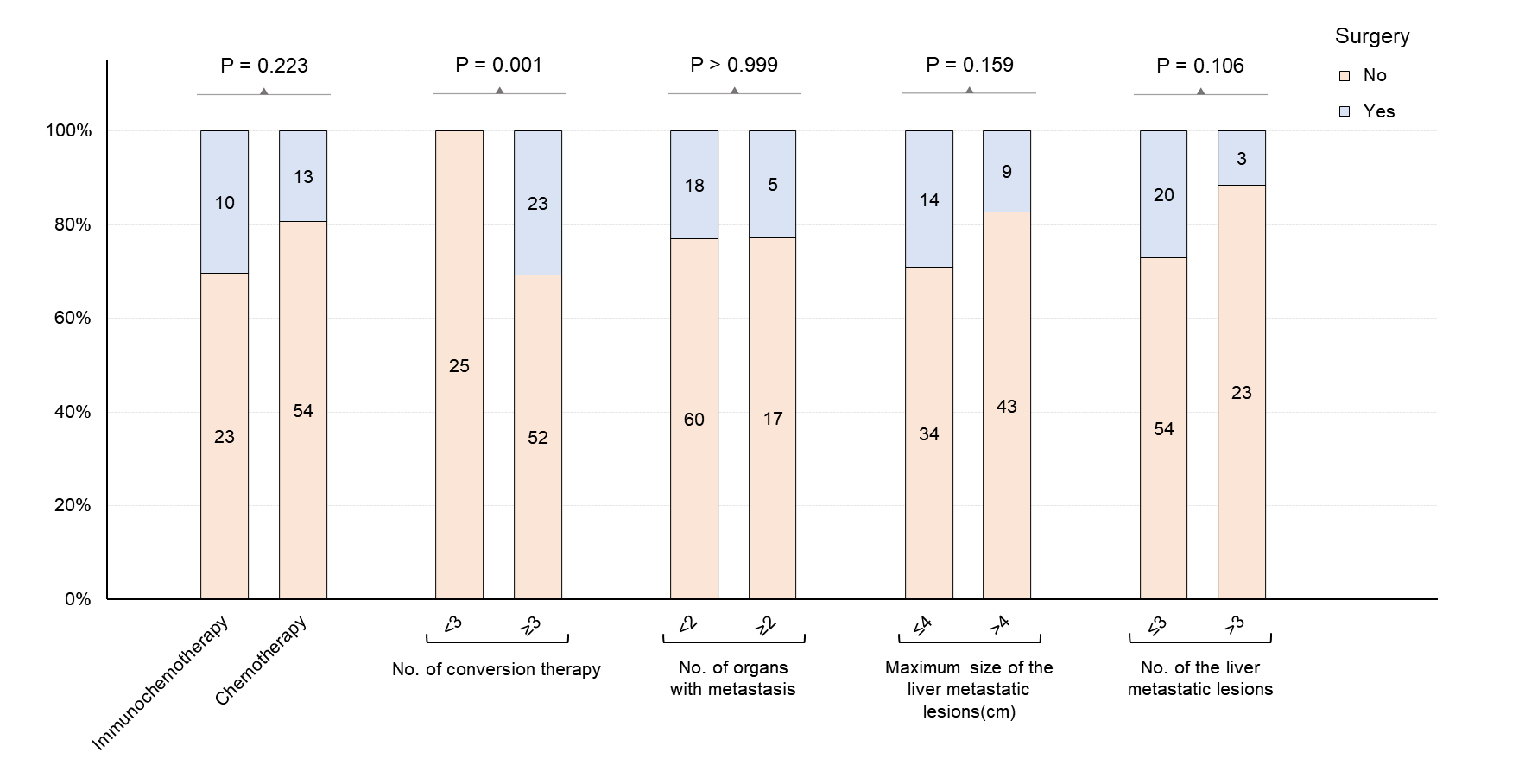


**eFigure 1.** Surgical conversion rate for the different groups


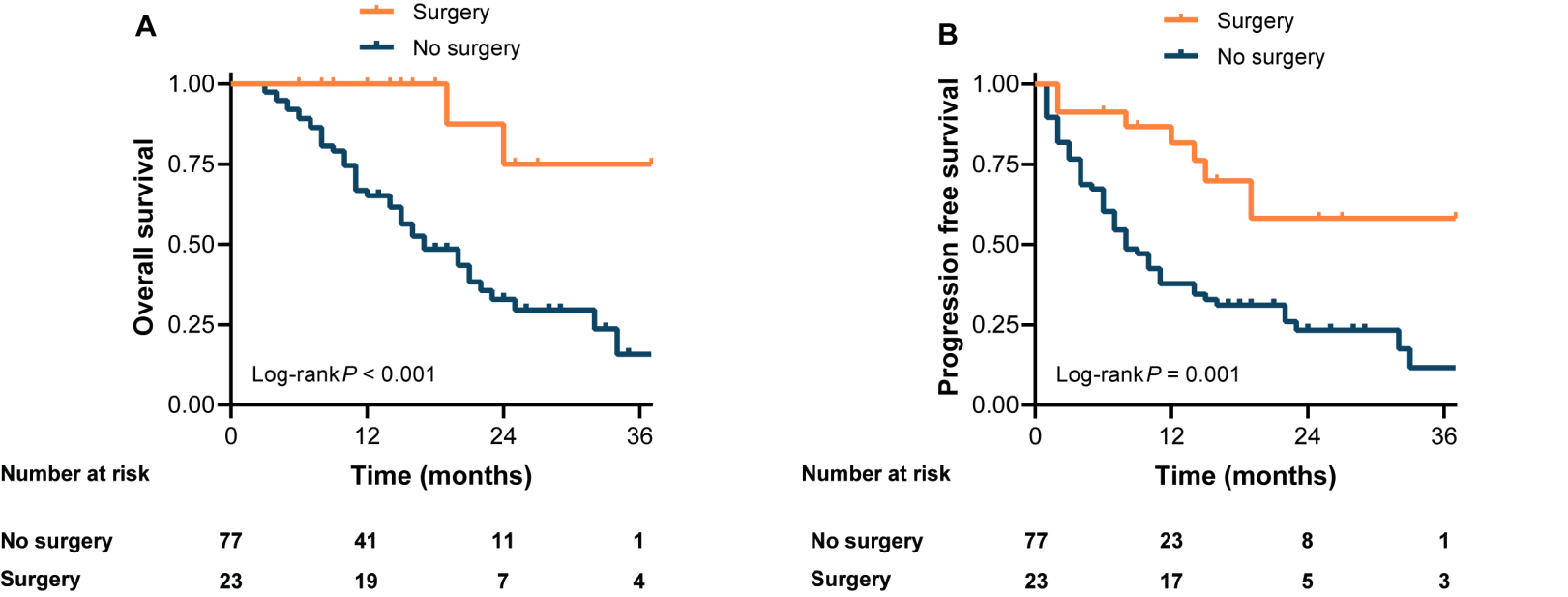


**eFigure 2.** the prognosis of different conversion therapy and surgery. A: overall survival; B: progress free survival.

**
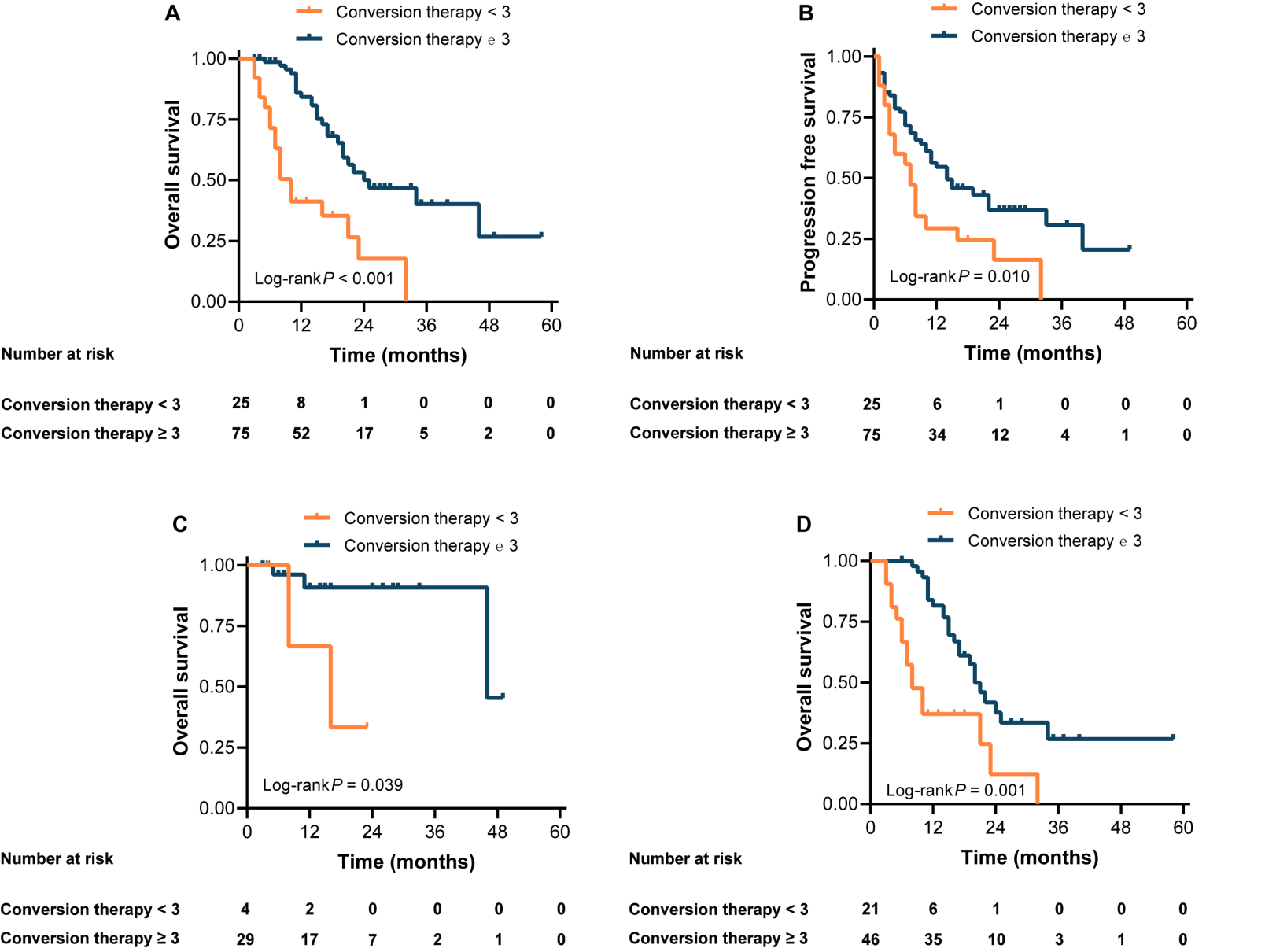
eFigure 3.** Survival analysis. A: Relationship between the number of conversion therapies and OS; B: Relationship between the number of conversion therapies and PFS. C: OS and number of conversion therapies in the immunochemotherapy group. D: OS and number of conversion therapies in the chemotherapy alone group (OS: overall survival, PFS: Progress free survival).
